# Effect of in situ simulation training for emergency caesarean section on maternal and infant outcomes

**DOI:** 10.1186/s12909-023-04772-6

**Published:** 2023-10-19

**Authors:** Yin Wang, Dehong Liu, Xiumei Wu, Chenmin Zheng, Xianxia Chen

**Affiliations:** 1 Department of Obstetrics and Gynecology, Anhui Province Maternity and Child Health Hospital, Hefei, China; 2 Anhui, China

**Keywords:** In situ simulation training, Obstetric rapid response team, Emergency caesarean section, Decision delivery interval, Maternal and infant outcomes

## Abstract

**Background:**

Emergency caesarean section (ECS) is an effective method for rapid termination of pregnancy and for saving maternal and foetal life in emergencies. Experts recommend that the interval from decision of operation to the decision to delivery interval (DDI) should be shortened as much as possible. Studies have shown that improving communication skills among staff by performing simulation drills shortens DDI, thus reducing the occurrence of adverse obstetric events and protecting maternal and child safety. In situ simulation (ISS) training is a simulation-based training approach for clinical team members conducted in a real-world clinical setting. In August 2020, Anhui Maternal and Child Health Hospital began ISS training on the rapid obstetric response team (RRT) in our hospital area for emergency caesarean section. This study aimed to investigate the effect of implementing in situ simulation training for emergency caesarean section on maternal and child outcomes by comparing maternal and child-related data on emergency caesarean section in two hospital areas.

**Methods:**

Data on cases of emergency caesarean delivery implemented in two hospital districts from August 2020 to August 2022 were collected: 19 in the untrained group and 26 in the training group. The two groups were compared concerning the interval from the decision of operation to the decision to delivery interval (DDI), the interval from the decision of operation to the initiation of skin incision, the interval from skin incision to the decision to delivery interval, and the neonatal situation.

**Results:**

Primary outcome comparison: The training group had a significantly shorter interval between the DDI compared to the untrained group (8.14 ± 3.13 vs. 11.03 ± 3.52, *P* = 0.006). Secondary outcomes comparison: The training group had a significantly shorter interval between the decision to cut skin compared to the untrained group (6.45 ± 2.21 vs. 9.95 ± 4.02, *P* = 0.001). However, there was no significant difference in the interval between cutting skin and infant delivery between the two groups (2.24 ± 0.08 vs. 2.18 ± 0.13, *P* > 0.05). Additionally, the Apgar score at 1 min after birth was higher in the training group compared to the untrained group (7.29 ± 2.38 vs. 6.04 ± 1.46, *P* < 0.05).

**Conclusions:**

The DDI for emergency caesarean section procedures can be significantly shortened, and neonatal Apgar scores at 1 min improved by implementing in situ simulation training for emergency caesarean section in obstetric rapid response teams. In situ simulation training is an effective tool for training in emergency caesarean section procedures and is worth promoting.

## Background

Emergency caesarean section (ECS) is an effective method for rapid termination of pregnancy and for saving maternal and foetal life in emergencies. Emergency cesarean sections are frequently associated with high maternal and neonatal mortality rates [1].

As a measure of standard of care, the decision-to-delivery interval of emergency caesareans has become an important determinant of perinatal outcomes [2]. Experts recommend that the interval from decision of operation to the decision to delivery interval (DDI) should be shortened as much as possible [3]. According to the American College of Obstetricians and Gynecologists, the interval between the decision to perform a caesarean section and delivery of the fetus should not exceed 30 min [4].

Studies have shown that improving communication skills among staff by performing simulation drills shortens DDI [5, 6], thus reducing the occurrence of adverse obstetric events and protecting maternal and child safety [7]. In situ simulation (ISS) training is defined as “simulating in the actual patient care setting or environment so as to achieve an extremely realistic level of fidelity” [8]. It is a simulation-based training approach for clinical team members conducted in a real-world clinical setting [8]. As well as improving the performance of new hospital units and protocols, in-situ simulation has been shown to be highly effective for identifying and correcting latent safety threats [9].

In August 2020, Anhui Maternal and Child Health Hospital began ISS training on the rapid obstetric response team (RRT) in our hospital area for emergency caesarean section. This study aimed to investigate the effect of implementing in situ simulation training for emergency caesarean section on maternal and child outcomes by comparing maternal and child-related data on emergency caesarean section in two hospital areas.

## Methods

### Clinical data

Our facility consists of two hospital districts located in different parts of the urban area, specifically referred to as our headquarters and western districts. The two districts exhibit similarities in terms of their size, facilities, and staffing. Since August 2020, regular in-situ simulation training for emergency cesarean section has been conducted for the obstetric Rapid Response Team (RRT) in our headquarters, whereas such training has not been organized in western districts. The training group consisted of emergency cesarean sections performed in the delivery room of our headquarters, while the non-training group comprised emergency cesarean sections performed in the delivery room of the western districts. According to guidelines established by our institutional system, indications for initiating an emergency cesarean section in the delivery room include umbilical cord prolapse, severe fetal distress, uterine rupture, placenta previa bleeding, amniotic fluid embolism, and other acute and critical situations that pose an imminent threat to the well-being of the mother and child. Prolonged deceleration lasting more than 5 min was defined as severe fetal distress [10]. The 24-h-duty staff in the delivery rooms of the two hospital areas included two first-line doctors, one general obstetric resident, one anaesthetist, one anaesthetic nurse, and one midwife (6–10). Paediatricians and second- and third-line physicians were on duty for 24 h in the hospital. In situ simulation training for emergency caesarean section on RRT for obstetrics in our hospital district has been organised regularly since August 2020. All cases in which ECs were implemented in the operating room of the delivery room of two hospitals from September 2020 to August 2022 were collected, resulting in a total of 47 cases, including 19 cases in the untrained group of the western hospital and 26 cases in the training group of our hospital area. Considering that the effect of training may not immediately affect clinical outcomes, 2 cases in the month of training initiation (August 2020) were not included in the study.

### In situ simulation training process

#### Upfront preparation

The medical office coordinated and established a simulation exercise team, and the director of the birthing center acted as the leader. Lectures were organised before simulation training to introduce knowledge related to emergency caesarean section procedures and in situ simulation training, including the delivery room environment, indications for emergency caesarean section procedures, and surgical process considerations. Training rounds occur semiannually. Each round lasted 4 weeks, began at 4 pm three weeks into the training period, and was completed by 3 sets of personnel each time. Training set: delivery room. Participating trainers: obstetricians, midwives, anaesthesiologists, and neonatologists of all shifts, divided into 12 groups, each containing one second-line physician (repeated grouping by ten attending and above physicians), one total obstetric stay (repeated grouping by two attending and above physicians), two residents, three midwives, one anaesthesiologist (repeated grouping by ten anaesthesiologists), one anaesthesia nurse (two anaesthesia nurses repeated grouping) and one neonatologist (four paediatricians repeated grouping). Attend to the individual roles and tasks of the personnel. All enrollees were required to participate in at least one in situ simulation training session. The team leader commanded and timed and third-line physicians (four obstetric ward directors) observed and hosted posttraining discussions and comments.

### Implementation process

A prolapsed cord was used as the subject for the scenario. A high-fidelity simulation was accomplished using a mannequin with a fetus and varying fetal heart tones to mimic a prolapsed cord. The first responder was the midwife, who noted the cord prolapse. The actual alarm bells on the unit were used to alert the other participants. Obstetricians, anesthetists, and pediatricians all responded, took on their usual roles, and performed their respective tasks. This included applying pressure on the fetal head, communicating with the family, initiating general anesthesia, beginning surgery, and resuscitation of the newborn.

### Postsimulation site evaluation and discussion

After on-site evaluation and discussion after simulation, the team members fill in the satisfaction questionnaire on site. The satisfaction questionnaire was designed and prepared by ourselves in combination with the actual situation of the hospital after consulting the literature [11], and the content put forward 6 questions centering on the simulated training:① Whether the training objectives are clear; ② Whether the training scenarios are related to clinical practice;③ whether the established learning objectives have been achieved;④ Whether the knowledge gained from this training can influence their clinical practice;⑤ Whether this training has enhanced their confidence in treating patients in crisis;⑥ Whether this training has given you more new knowledge and skills. Participants were asked how much they agreed with this question using a 5-point Likert scale (strongly agree, agree, fair, disagree, strongly disagree). After the completion of the questionnaire collection, on-site discussion was conducted. First, team members made self-evaluation and reflected on their own and team performance. Then, third-line doctors made comments, including the operation completion degree, cooperation degree and DDI of each member. Finally, the improvement contents were proposed.

### Outcome measures

Primary outcomes: DDI; Secondary outcomes: trainees’ satisfaction with the training, demographics of both groups, decision to incision interval, incision to fetal delivery interval, indications for cesarean section, operation time, intraoperative bleeding, neonatal birth weight, 1 min Apgar score, 5 min Apgar score and length of patient stay.

### Statistical methods

The data were statistically analysed by IBM SPSS 23.0 software, and the data were measured as mean ± standard deviation. Normally distributed data were compared by t test, and nonnormally distributed data were compared by rank sum test. Count data were compared with χ2 or Fisher’s exact test. *P* < 0.05 was considered statistically significant.

## Results

### Trainee satisfaction with training effectiveness

A total of 92 individuals participated in the training, and 92 questionnaires were distributed in total, with a recovery rate of 100%. The results showed that 100% of the trainees believed that the training objectives were clear and the training scenarios were relevant to clinical practice, 97.83% believed that they met the established learning objectives and believed that the knowledge gained from this training could affect their own clinical practice, 95.65% believed that the training enhanced their confidence in treating patients in the event of a crisis, and 98.91% believed that they benefited significantly (Table 1).

**Table 1:** Trainee satisfaction with training

	Strongly agree n(%)	Agree n(%)	General n(%)	disagree n(%)	Strongly disagree n(%)
The goals of training are clear	92 (100.00)	0 (0.00)	0 (0.00)	0 (0.00)	0 (0.000
Training scenarios and relevance to clinical practice	92 (100.00	0 (0.00)	0 (0.00)	0 (0.00)	0 (0.00)
The established study objectives were met	90 (97.83)	2 (2.17)	0 (0.00)	0 (0.00)	0 (0.00)
The knowledge gained from this training can influence one’s own clinical practice	90 (97.83)	2 (2.17)	0 (0.00)	0 (0.00)	0 (0.00)
This training enhanced one’s own confidence in treating patients in the event of a crisis	88 (95.65)	4 (4.35)	0 (0.00)	0 (0.00)	0 (0.00)
Training lets yourself harvest more new knowledge and skills	91 (98.91)	1 (1.09)	0 (0.00)	0 (0.00)	0 (0.00)

### Comparison of demographics between the two groups

The untrained group with 19 cases accounted for 1.32% (19/1440) of caesarean deliveries in delivery rooms during the same period, and the trained group with 26 cases accounted for 1.37% (26/1900) of caesarean deliveries in delivery rooms during the same period. The demographics of the two groups were compared, and there was no significant difference (*P* > 0.05) (Table 2).

**Table 2:** Comparison of demographics between the two groups

Group	Untrained group	Training group	t Value	*P* Value
	(*n*=19)	(*n*=26)		
Years (years)	24.1 ± 3.8	26.4 ± 5.1	-1.656	0.105
BMI(kg/m^2^)	27.2 ± 3.1	26.3 ± 2.2	1.140	0.260
Gestational weeks (wk)	37.1 ± 3.8	37.0 ± 4.6	0.077	0.939
Gravidity	2.3 ± 1.3	2.2 ± 1.1	0.279	0.782
Parity	1.2 ± 1.1	1.0 ± 0.7	0.745	0.460

### DDI comparison between the two groups

The interval between the DDI and decision to cut the skin was significantly shorter in the training group than in the untrained group (*P* < 0.05), and the interval between cutting the skin and foetal delivery was not significantly different between the two groups (*P* > 0.05) (Table 3).

**Table 3:** Comparison of the DDI between the two groups

Group	Untrained group	Training group	t value	*P* Value
	(*n*=19)	(*n*=26)		
DDI(min)	11.03 ± 3.52	8.14 ± 3.13	2.903	0.006
Decision to cut skin interval (min)	9.95 ± 4.02	6.45 ± 2.21	3.742	0.001
Interval between skin resection and infant delivery (min)	2.18 ± 0.13	2.24 ± 0.08	-1.913	0.062

### Comparison of Surgery, neonatal condition and length of hospital stay between the two groups

There were no significant differences between the two groups in the indication for surgery, operation time, blood loss, neonatal birth weight and length of hospital stay (*P* > 0.05). The Apgar scores at one minute of neonatal birth were higher in the training group than in the untrained group, with statistically significant differences (*P* < 0.05). There was no statistically significant difference observed in the incidence of new-borns with a 5-minute Apgar score below 7 between the two groups (Table 4).

**Table 4:** Comparison of surgery, neonatal condition and length of hospital stay between the two groups

Group		Untrained group	Training group	t/χ^2^-Value	*P* Value
		(*n*=19)	(*n*=26)		
Indications for caesarean section ^a^	foetal distress	7(36.8%)	9(34.6%)		1.000
	Umbilical cord prolapse	10(52.6%)	14(53.9%)		
	Abruptio placenta	2(10.6%)	2(7.7%)		
	Negligible transverse position	0(0%)	1(3.8%)		
Operative time(min)		53.1 ± 11.3	52.1 ± 11.7	0.287	0.775
Blood loss(ml)		435 ± 130.1	415 ± 89.6	0.611	0.544
birth weight(g)		3401.7 ± 300.1	3389 ± 320.3	0.135	0.893
Apgar score at 1 min		6.04 ± 1.46	7.29 ± 2.38	2.204	0.049
5-minute Apgar score < 7		1(5.0%)	2(7.7%)	0.134	0.714
Length of hospital stay		6.17 ± 2.31	7.05 ± 2.27	-1.275	0.209

^a^ The minimum theoretical frequency of indications for caesarean delivery, 0.4222222, < 1, was statistically tested using Fisher’s exact probability

## Discussion

 In theory, ameliorating DDIs and shortening the time that a foetus is subjected to acute stress should improve outcomes. The standard of 30 min for DDI is routine practice [12]. Control of DDI within 5 min is recommended for moribund caesarean Sect. [13]. However, studies exploring the relationship between DDIs and maternal and foetal outcomes were conflicting. Some studies showed that shortening DDIs in the ECS could improve early neonatal outcomes [14] while other studies concluded that the neonatal outcomes were not necessarily adversely affected when DDIs exceeded 30 min [15]. In this study, along with the obvious shortening of the DDI, the Apgar scores of newborns at 1 min after birth in the training group were higher than those in the untrained group, affirming the importance of shortening the DDI. In addition, the DDI of cases in this study, whether in the untrained or trained group, was significantly shorter than in other studies [16]. This may be related to the fact that members of the ECS team at our institution are on call 24 h, midwives act directly as instrument nurses, and the surgeon (both attending physicians and second-line physicians) are skilled and can control the skin incision to infant delivery interval to within 3 min. It may also be related to the indication for surgery when implementing ECs, the absence of a scarred uterus in the study, and other cases of significantly lower DDI [5].

Shortening DDIs is challenging in practical situations. A skilled and well-trained obstetric RRT is needed, and team members include obstetrics, anaesthesiology, neonatologists, midwives, anaesthesia nurses, and other staff [16]. However, ECS is a rare clinical event, and ECS in our hospital accounts for only 1.41% (47/3340) of transferred caesarean deliveries in the delivery room during the same period. It is not realistic to rely on the cumulative experience of RRT in obstetrics in clinical practice. Research has shown that simulation training is an effective tool in crisis resource management teaching; can improve team confidence, behaviour, and task knowledge; and can effectively prevent errors [17]. ISS training was conducted in a real-world clinical setting. Studies have confirmed that ISS training of the ECS for obstetric RRT can reduce the interval time from diagnosis to admission to the operating room [16, 18] as well as DDI [16, 19] and increase the proportion of cases achieving the target time (within 30 min) [20, 21]. In this study, it was similarly found that after training, the DDI was significantly shorter, and the reason for the shorter DDI was attributed to the shorter interval from the decision to skin cutting, confirming the effectiveness of ISS training. ISS training is an effective tool for ECS training. The first is the high compliance of the participating personnel, and studies have shown that trainees believe such simulation is more realistic and are therefore more willing to participate [22]. Second, ISS training not only improves teamwork and clinical practice but also helps identify barriers that exist in real-life scenarios. Hamman et al. [23] found from 40 field simulations that personnel did not know enough about the identification of items, and it was difficult to find the required drugs and equipment during actual operation. Postidentification targeted design solutions and interventions, resulting in increased work productivity, which non-ISS training cannot do. Then, other health care workers in the field who are not involved in ISS training can act as observers and have the opportunity to improve teamwork skills by observing the simulation and participating in debriefing, even if they are not involved in the simulation in person [24].

### Limitations

Our study has some limitations that we would like to highlight: first of all the diagnosis of neonatal asphyxia could not be established with the detection of umbilical artery pH because of conditional limitations, and the lack of long-term follow-up results precluded drawing results on long-term neonatal outcomes such as the incidence of cerebral palsy. Moreover, the interval from the appearance of an abnormal condition to the decision to perform ECS is strongly associated with foetal prognosis [25], but this time is not constant and depends on the decision-making ability of the obstetrician, which seems to be a major but difficult limitation to avoid.

## Conclusions

In conclusion, the DDI for emergency caesarean section procedures can be significantly shortened, and neonatal Apgar scores at 1 min improved by implementing in situ simulation training for emergency caesarean section in obstetric rapid response teams, which is highly recognised by the trainees. In situ simulation training is an effective tool for training in emergency caesarean section procedures and is worth promoting.

## Data Availability

The datasets used and/or analysed during the current study are available from the corresponding author on reasonable request.

## Abbreviations

ECS: Emergency caesarean section
DDI: Decision to delivery interval
RRT: Rapid obstetric response team
ISS: In situ simulation
